# Intraoperative risk factors for major complications after oesophagectomy: the surgical Apgar score

**DOI:** 10.1093/icvts/ivac111

**Published:** 2022-05-06

**Authors:** Lucio Cagini, Silvia Ceccarelli, Umberto Bracale, Valentina Tassi

**Affiliations:** 1 Department of Thoracic Surgery, Ospedale del Mare, ASL Napoli1 Centro, Naples, Italy; 2 Department of Thoracic Surgery, Azienda Ospedaliera S Maria della Misericordia, Perugia, Italy; 3 University Federico II of Naples, Naples, Italy; 4 Thoracic Surgery Unit, Azienda Ospedaliera Santa Maria, Terni, Italy

**Keywords:** Oesophagectomy, Complications, Pneumonia, Apgar score, Risk factors

To date, numerous studies have sought to identify reliable predictors of developing postoperative complications; demanding challenges for general thoracic surgeons in their daily practice [1, 2]. Oesophagectomy, both for benign and malignant diseases, is a complex surgical procedure characterized by relevant rates of mortality and morbidity, along with the worse quality of life [3, 4]. Although current postoperative mortality is <5% at high-volume centres, major complications remain significant, from 26% to 31% [5]. These translate into longer hospital stays and higher overall patient costs, which increase according to their severity [6]. Finally, the overall morbidity associated with the operation has not been significantly reduced yet, even with the minimally invasive approach, thus suggesting that the pathogenesis of complications associated with oesophagectomy is multifactorial [7].

Efforts have been made to identify patient-related prognostic factors for major comorbidities and anastomotic leakage. In a recent meta-analysis, van Kooten and colleagues [5] found male sex [odds ratio (OR) 4.50; 95% confidence interval (CI) 1.21–16.64; *P* = 0.02], cardiac comorbidity (OR 1.53; 95% CI 1.25–1.87; *P* = 0.01) and diabetes (OR 1.93; 95% CI 1.14–3.26; *P* = 0.01) to be significantly associated with major complications. Moreover, 9 risk factors for anastomotic leakage have also been identified, with the renal disease being the most prominent.

Although much attention has been centred on preoperative conditions, the present article by Zheng *et al.* [8] focuses on intraoperative risk factors which might be useful for clinicians to decrease complications. In ‘Surgical Apgar score could predict complications after oesophagectomy: a systematic review and meta-analysis’, the authors sought to shed light on the role of surgical Apgar score (SAS) in predicting adverse events after oesophagectomy. Concerning the secondary outcomes, the meta-analysis of 4 studies concluded that SAS could predict the incidence of pulmonary complications (OR = 2.32, 95% CI: 1.61–3.36, *P* < 0.001); whereas the association between SAS and 5-year survival rate, as well as perioperative mortality, was not investigated since they were reported only in one study each.

This stimulating paper helps to clarify the role of the SAS in predicting major complications after oesophagectomy. Indeed, since Gawande first applied the SAS score calculated on 3 intraoperative variables, estimated blood loss, lowest mean arterial pressure and lowest heart rate, several studies on this topic have been carried out leading to inconsistent results [9]. While it has been proven to be an effective tool in predicting complications after surgery in some urological, gynaecological and digestive operations, regarding oesophagectomy for cancer, this has failed to be confirmed. Why should surgeons be interested in the findings of this meta-analysis?

The implications of these reported results include that we might be able to better screen for patients who are at a high risk of developing postoperative complications. With this exceedingly simple scoring system, clinicians could optimize the individual perioperative management by applying closer postoperative surveillance or delayed enteral feeding in high-risk patients, so to decrease the probability of postoperative complications. If so, this would have an impact on shortening hospital stays and reducing overall patient costs.

Future high-quality, prospective studies aimed at confirming these findings would be beneficial in assessing the usefulness of the SAS in reliably predicting complications after oesophagectomy. Zheng *et al.* are to be congratulated on their contribution to this field of understanding. Their findings will certainly prove to be most beneficial to the surgical community if confirmed in larger studies.

**Conflict of interest:** none declared.
